# Characterization of Poly(3-hydroxybutyrate) (P3HB) from Alternative, Scalable (Waste) Feedstocks

**DOI:** 10.3390/bioengineering10121382

**Published:** 2023-11-30

**Authors:** Rogerio Ramos de Sousa Junior, Fabiano Eduardo Marques Cezario, Leonardo Dalseno Antonino, Demetrio Jackson dos Santos, Maximilian Lackner

**Affiliations:** 1 Center for Engineering, Modeling and Applied Social Sciences, Federal University of ABC, Santo André 09210-580, Brazil; rogerio.sousa@ufabc.edu.br (R.R.d.S.J.); fabiano.cezario@ufabc.edu.br (F.E.M.C.); leonardoantonino@hotmail.com (L.D.A.); 2 Circe Biotechnologie GmbH, Kerpengasse 125, 1210 Vienna, Austria; m.lackner@circe.at

**Keywords:** poly(3-hydroxybutyrate) (P3HB), cyanobacteria, methanotrophs, gas fermentation, bioplastics, mechanical properties, thermal properties

## Abstract

Bioplastics hold significant promise in replacing conventional plastic materials, linked to various serious issues such as fossil resource consumption, microplastic formation, non-degradability, and limited end-of-life options. Among bioplastics, polyhydroxyalkanoates (PHA) emerge as an intriguing class, with poly(3-hydroxybutyrate) (P3HB) being the most utilized. The extensive application of P3HB encounters a challenge due to its high production costs, prompting the investigation of sustainable alternatives, including the utilization of waste and new production routes involving CO_2_ and CH_4_. This study provides a valuable comparison of two P3HBs synthesized through distinct routes: one via cyanobacteria (*Synechocystis* sp. *PCC 6714*) for photoautotrophic production and the other via methanotrophic bacteria (*Methylocystis* sp. *GB 25*) for chemoautotrophic growth. This research evaluates the thermal and mechanical properties, including the aging effect over 21 days, demonstrating that both P3HBs are comparable, exhibiting physical properties similar to standard P3HBs. The results highlight the promising potential of P3HBs obtained through alternative routes as biomaterials, thereby contributing to the transition toward more sustainable alternatives to fossil polymers.

## 1. Introduction

Plastics are produced in the order of 400 million tons/year, mostly from fossil re-sources, contributing to environmental challenges such as inefficient and limited end-of-life management and the release of micro- and nano-plastics into the environment [1,2]. This inefficiency arises from the predominantly linear (unidirectional) use of plastics. Only a small fraction of 9% of all plastics is recycled [3], despite decades of efforts to collect and recycle more plastic materials, especially through the mechanical recycling of thermoplastics [4]. The low value of materials, their diversity regarding polymers and contained additives, and the contamination of post-consumer waste make collection and recycling costly and technically challenging [5]. An estimated up to 8% of all petroleum is used to produce plastics, resulting in greenhouse gas emissions throughout the life cycle, beginning with production and often ending with waste incineration, since more and more countries are implementing landfill bans for these material [6]. As the adverse environmental effects of traditional plastics become more apparent, there is a growing imperative to explore sustainable alternatives, where biobased and biodegradable polymers can play a key role, in addition to reduction, reuse and recycling as well as, e.g., extended producer responsibility laws throughout the value chain [7,8]. Polyhydroxyalkanoates (PHAs), with their eco-friendly nature and versatile properties, are emerging as promising alternatives to conventional plastics [9].

PHAs are naturally occurring polyesters formed by microorganisms as energy and reducing power storage [10,11]. Additionally, they exhibit thermoplastic properties and are biodegradable. Although the term “biodegradable” is subject to various definitions, PHAs demonstrate biodegradability in both aerobic and anaerobic systems at ambient and below-ambient temperatures, including in marine environments [12]. This characteristic makes them particularly interesting for micro- and nano-plastics avoidance. 

The most common PHA is poly(3-hydroxybutyrate) (P3HB) [13]. While P3HB is a rather brittle material, its copolymers (e.g., poly(3-hydroxybutyrate-co-3-hydroxyvalerate) (PHBV)) and blends are softer and more flexible [14]. Other types of PHAs, such as polyhydroxyhexanoate (PHHx) or polyhydroxyalkanoate (PHO), are commercially available, and additional types, like medium-chain length PHA (mcl-PHA), are under development, potentially replacing elastomers in the future [13]. 

It is estimated that PHAs have the potential to replace up to 90% of all conventional plastics, especially commodity materials, addressing the persistent issue of microplastics associated with classic non-degradable plastics [15]. Despite its initial brittleness, P3HB distinguishes itself among PHAs by displaying physical characteristics similar to polypropylene (PP) [16,17], presenting significant potential applications ranging from packaging to diverse biomaterial uses [16,18,19,20,21,22]. The application of P3HB as biomaterial is crucial for various biomedical purposes, including drug delivery, tissue engineering, and medical apparatus fabrication, owing to its biocompatibility, biodegradability, and versatile properties [23]. Biocompatibility is vital for in vivo devices, avoiding immunological responses, while biodegradability eliminates the need for additional surgical removal [21]. Yao et al. [24] fabricated films of P3HB and PHBV through solvent casting and electrospinning, exploring their potential as scaffolds for vascular tissue engineering. The electrospun films demonstrated a nano-porous microstructure, exhibiting promising physical properties for vascular engineering applications, in addition to low cytotoxicity and high biocompatibility. Esposti et al. [25] investigated highly porous scaffolds made of P3HB and hydroxyapatite for bone tissue engineering, evaluating both in situ and ex situ approaches for hydroxyapatite synthesis. The results suggest that the in situ approach was more effective in incorporating hydroxyapatite and preserving porosity, promising mechanical and biological properties for applications in bone tissue engineering.

One constraint on the extensive utilization of P3HB is the elevated costs associated with obtaining it, stemming from substrate costs, the scale of production, and process efficiency [21]. Traditional P3HB production relies on fermentation processes, utilizing sugars from sources like sugarcane and starch [26]. Innovative approaches have sought to enhance sustainability and reduce costs by utilizing waste feedstocks, such as sewage, as a low-cost nutrient source [15]. Efforts are underway to produce PHA not only from primary agricultural products but also from side streams and waste streams of renewable raw materials. This study explores P3HB derived from two alternative production routes, one utilizing CO_2_ (converted into P3HB by cyanobacteria) and the other based on methane (with methanotrophs facilitating P3HB production). Both gases are abundantly available from biomass waste and various renewable resources, providing a potential means to scale up P3HB production and replace conventional plastics. CO_2_ can be sourced from waste incineration plants, cement plants, or through direct air capture via cyanobacteria [27]. Renewable CH_4_ is accessible through biogas, landfill gas, and processes like power to gas (P2G) [28].

While these advancements broaden the scope of PHA production, questions arise regarding the impact of production methods on the physical and chemical properties of resulting polymers [29]. Various studies, such as that by Pradhan et al. [30], have obtained P3HBs from *B. megaterium* and *C. necator* and evaluated their physical properties in comparison to a standard P3HB, with a focus on thermal behavior. In general, the P3HBs exhibited similar properties but displayed noticeable variations in crystallinity, with 44% and 23% for the materials derived from *B. megaterium* and *C. necator*, respectively. Domínguez-Díaz et al. [31] conducted an analysis of P3HBs derived from cultures of *A. vinelandii* wild type strain OP and mutant strains with specific gene mutations, with molecular weights ranging from 0.16 × 10^6^ Da to 2 × 10^6^ Da. They observed a correlation between molecular weight and physical properties up to the threshold of 1 × 10^6^ Da. Beyond this threshold, a reduction in crystallinity, possibly due to molecular entanglement effects, led to a decrease in elastic modulus.

Besides the higher costs associated with obtaining P3HB compared to fossil polyolefins on a large scale, there are additional challenges for its extensive adoption. These include a narrow processing window, low toughness, and brittleness [14]. Regarding mechanical brittleness, molded P3HB undergoes a detrimental aging process when stored at room temperature, resulting in progressive embrittlement and a drastic reduction in elongation at break. During the aging process, two phenomena contribute to P3HB embrittlement: secondary crystallization and physical aging [32]. In this context, understanding the correlation between the source of P3HB and its physical properties is essential to optimize applications and overcome these limitations.

This study conducts a comparative analysis of two P3HBs, denoted as P3HB1 and P3HB2, synthesized through different production methods. The first method involves photoautotrophic production utilizing cyanobacteria (*Synechocystis* sp. *PCC 6714*), and the second one is chemoautotrophic growth using methanotrophic bacteria (*Methylocystis* sp. *GB 25*). The main objective is to thoroughly characterize the P3HBs obtained via different production routes, analyzing their physical properties. Additionally, we aim to compare these properties with those of standard P3HBs, focusing on their potential for various bioapplications and providing valuable insights into the distinct properties of P3HBs synthesized through alternative routes. In the context of the growing demand for sustainable alternatives to traditional plastics, this research contributes to advancing our understanding of P3HB as a biomaterial with versatile production routes, highlighting its increasing potential for bioapplications.

## 2. Materials and Methods

### 2.1. Materials

Two P3HBs were produced using different methods: photoautotrophically through *Synechocystis* sp. *PCC 6714* and chemoautotrophically through *Methylocystis* sp. *GB 25*, both in pure culture. The former microorganisms derive their carbon from CO_2_ and energy from sunlight, while the latter draw their carbon and energy from methane. CO_2_ can be taken from the air (natural assimilation) or from a point source such as flue gas (after cleaning and cooling), and CH_4_ is accessible through biogas and synthetic natural gas (SNG) from syngas and from CO_2_ (plus H_2_) via methanation (compare the power to gas concept). In this work, CO_2_ from room air (direct assimilation by the cyanobacteria) was deployed, while the CH_4_ was used as a bottled gas since fermentation was carried out at a 30 L lab scale, which requires comparatively low quantities of feed gasses. The CH_4_ was bottled at a local SNG fueling station in lower Austria (gas composition 98.5% CH_4_, 1.5% CO_2_, H_2_S < 20 ppm). The oxygen was taken from room air (not enriched) after passing a sterile filter. A higher mass transfer could have been achieved by using pure CO_2_ and pure O_2_, but this was not optimized in the current experiments.

The two distinct P3HBs were extracted from biomass through a process involving acetone/ethanol for precipitation, followed by vacuum drying. This method eliminates the need for chlorinated solvents like chloroform or dichloromethane. The obtained P3HBs were in the form of irregular sheets, which were subsequently ground into powder. The extraction efficiency was determined to be >90% (by mass). Table 1 presents the main characteristics of each P3HB.

### 2.2. Experimental Procedure

The P3HBs were characterized as received (unprocessed) in order to obtain the intrinsic properties of these materials and, after processing with injection molding, to evaluate the crystalline structure and mechanical behavior of the polymeric materials. For the latter approach, the materials were characterized over time: immediately after processing (0 h), 6 h, 24 h, 72 h, 7 d, 14 d, and 21 d, as P3HB is known to crystallize over several days and even weeks, thereby changing its properties markedly.

### 2.3. Injection Molding Processing

The samples for tensile testing were obtained through injection molding using the micro-injector molder IM12 Xplore (Sittard, The Netherlands). The injection process was conducted at 175 °C, with an injection pressure of 6 bar and a total duration of 30 s. Samples for XRD analysis were obtained from the test specimens used for the tensile test and were analyzed at approximately the same position on the sample surface.

### 2.4. Characterizations

#### 2.4.1. ^1^H Nuclear Magnetic Resonance

The chemical structure of both P3HBs was accessed via proton nuclear magnetic resonance (^1^H NMR) analyses, which were performed on a Bruker Avance 500 MHz spectrometer (Bruker, Billerica, MA, USA) operating at 21 °C. The polymer solutions of ca. 10 mg mL^−1^ in chloroform-d_1_ were used. The acquisition parameters employed were: 1.6 s acquisition time; 1.0 s recycle delay, spectra width of 10,302 Hz; 16 scans; 32,000 points; and a free induction decay (FID) resolution of 0.63 Hz.

#### 2.4.2. Size Exclusion Chromatography

The number-average molecular weight (M_n_), mass-average molecular weight (M_w_), and polydispersity index (M_w_/M_n_) of each P3HB sample were determined using size exclusion chromatography (SEC) employing a Viscotek GPCmax VE2001 instrument (Malvern Panalytical, Worcestershire, United Kingdom) equipped with three columns (Shodex K-802, K-803, and K804) set at 40 °C, and chloroform as the eluent with a flow rate of 0.5 mL min^−1^. Polystyrene standards with molecular weight ranging from 935 to 1,790,000 g mol^−1^ were utilized to establish the relative molecular weight of the samples. P3HB samples were solubilized in chloroform (0.5 mg mL^−1^).

#### 2.4.3. Differential Scanning Calorimetry

Differential scanning calorimetry (DSC) measurements were performed using a DSC Q200 instrument (TA Instruments, Waltham, MA, USA). The first heating cycle was carried out from room temperature to 200 °C in order to erase the thermal history of the polymers, followed by cooling cycles to −40 °C and re-heating to 210 °C. All heating and cooling cycles were conducted at a rate of 10 °C min^−1^ under an inert nitrogen atmosphere. The crystallinity index via DSC (*X_DSC_*) was calculated according to Equation (1):

(1) $$X_{DSC}\left(\%\right)=\frac{{\Delta H}_{m}}{{\Delta H}_{m}^{0}}\times 100$$

where ${\Delta H}_{m}$ is the P3HB melting enthalpy in the sample and ${\Delta H}_{m}^{0}$ is the melting enthalpy for 100% crystalline P3HB (${\Delta H}_{m}^{0}$ = 146 J g^−1^) [33].

#### 2.4.4. X-ray Diffraction

The crystalline structure of P3HBs was evaluated using X-ray diffraction (XRD) with a D8 Discover diffractometer (Bruker, Billerica, MA, USA), equipped with a Cu Kα source (λ = 1.54 Å). The diffraction patterns were collected by scanning an angular range from 10° to 30°, with a step size of 0.02°, at a voltage of 40 kV and a current of 40 mA. The Fityk software (version 1.3.1) was employed for data analysis. The XRD crystallinity index (*X_XRD_*) was calculated using Equation (2):

(2) $$X_{XRD}\left(\%\right)=\frac{A_{c}}{A_{t}}\times 100$$

where $A_{c}$ is the sum of the areas under the crystalline peaks and $A_{t}$ is the total area of the diffraction profile.

#### 2.4.5. Tensile Test

Tensile tests were performed on a model 3369 universal mechanical testing machine (Instron, Norwood, MA, USA), equipped with a 50 kN load cell and a noncontacting video extensometer (AVE-Instron), in order to obtain mechanical properties such as elastic modulus (E), ultimate tensile strength (σ_max_), and elongation at break (ε_max_). The injection-molded specimens conform to the ISO 527-2 standard [34]. The tests were conducted at a crosshead speed of 2 mm min^−1^ using a gauge length of 20 mm.

## 3. Results and Discussion

### 3.1. Characterization of P3HB

**Figure 1:**
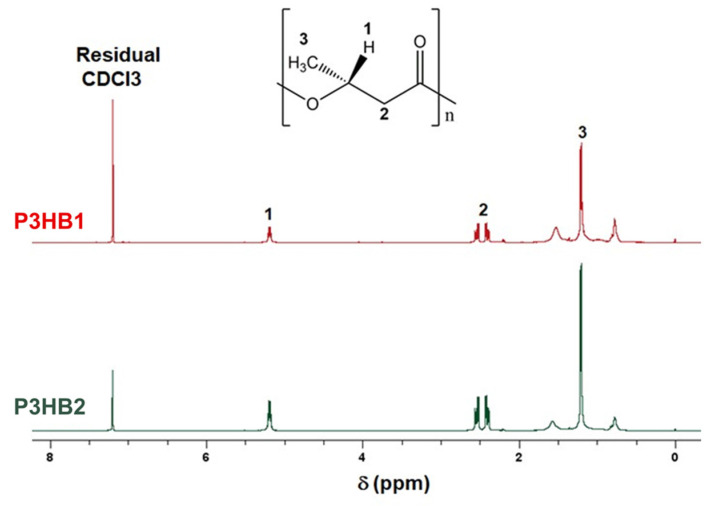
^1^H NMR spectra of P3HB1 and P3HB2.

The chemical structure of the two P3HB samples was investigated via ^1^H NMR analyses. The ^1^H NMR spectra of P3HB1 and P3HB2 are shown in Figure 1. Similar signals were observed for both samples. Four intense peaks were verified: (i) a singlet at δ = 7.2 ppm, corresponding to deuterated chloroform (CDCl_3_); (ii) a doublet at δ = 5.2 ppm; (iii) a multiplet at δ = 2.7–2.3 ppm; and (iv) a doublet at δ = 1.2 ppm. The peaks (ii), (iii), and (iv) are characteristic for P3HBs and were also observed in others works [35,36,37]. They are associated with, respectively, -CH, -CH_2_, and -CH_3_ protons found in P3HB monomers (hydroxybutyrate), as indicated in Figure 1. The result reveals that both investigated samples have identical chemical structures, which was expected (homopolymers of P3HB).

The molecular weight values of the P3HBs were determined via SEC analysis and are presented in Table 2. Both P3HBs exhibit similar M_w_ values, approximately 1 × 10^6^ Da, falling within the common range reported for this material, ranging from 0.2 × 10^6^ to 3 × 10^6^ Da [38]. Additionally, these values usually confer physical properties suitable for applications as thermoplastics and demonstrate a high elastic modulus [31,38].

### 3.2. Thermal Properties

**Figure 2:**
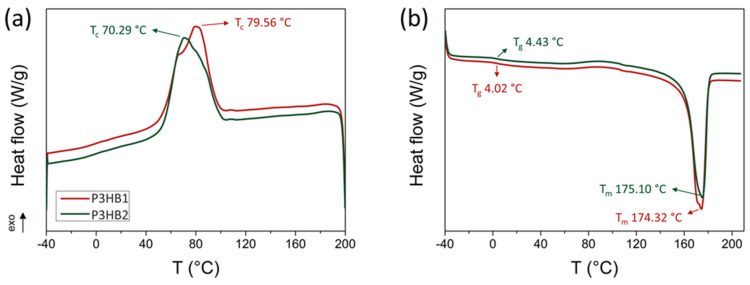
DSC curves of the (**a**) cooling and (**b**) second heating scans.

The DSC technique is the most widely used method to evaluate the thermal properties of a polymer, including P3HB [39]. Both P3HBs were analyzed in their as-received state (unprocessed). Figure 2 displays the cooling and the second heating cycles (Figure 2a and Figure 2b, respectively). The main thermal parameters obtained are presented in Table 3.

P3HB1 exhibits a higher crystallization temperature peak (T_c_) compared to P3HB2, measuring 79.56 °C and 70.29 °C, respectively. However, both polymers have the same crystallization temperature range. 

During the reheating scan (Figure 2b), the thermal behavior of both P3HBs is similar. The glass transition temperature (T_g_) is observed at approximately 4 °C, which falls within the 0 to 6 °C range reported in the literature [40,41,42]. The melting process displays a prominent peak, known as the melting temperature (T_m_), which reaches 174 °C for P3HB1 and 175 °C for P3HB2, respectively. These melting temperature values align with the observed range for unmodified P3HBs as reported in the literature [40,42]. Furthermore, *X_DSC_* values were calculated between 63 and 64%.

Thus, it is possible to affirm that the P3HB samples evaluated in this study are similar in terms of thermal properties obtained by DSC and comparable to standard P3HBs reported in the literature. Although there is a slight variation in the T_c_ peak, which may be related to the higher M_w_/M_n_ value of P3HB2, even though both have the same molecular weight (M_w_). Domínguez-Díaz et al. [31] reported that T_m_ and T_c_ are minimally affected by the molecular weight of P3HB, except for very high molecular weights (M_v_ > 1 × 10^6^ Da), with T_c_ demonstrating greater sensitivity (variation from 97 to 111 °C in a non-linear range from low to high molecular weight). Larger variations in these properties may be related to the restricted mobility of the amorphous phase of P3HB, leading to greater variations in T_c_ and, especially, in the values of T_g_ and crystallinity [30], which were not observed in the analyzed materials due to the similarity of M_w_. It is important to emphasize that P3HB is a semicrystalline polymer with high T_m_ values and crystallinity as characteristic [33]. This characteristic is of interest in bioapplications, where the T_m_ values (174–175 °C) exceed body temperature, as it is necessary for mechanical stability, while T_g_ in the order of 4 °C provides the amorphous phase with rubbery behavior under physiological conditions [22].

### 3.3. Crystalline Structure

**Figure 3:**
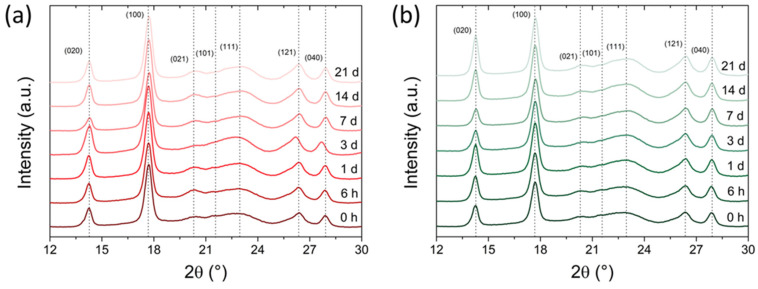
XRD of (**a**) P3HB1 and (**b**) P3HB2 at different aging times (0 h up to 3 weeks) at room temperature.

XRD analyses were performed on both P3HBs to evaluate the crystalline structure and the evolution of crystallinity degree over time. Figure 3 presents the diffractograms for P3HB1 and P3HB2 (Figure 3a and Figure 3b, respectively). From the diffractograms, two prominent peaks can be observed at 2θ = 14.3° and 17.7°, while less intense peaks are observed at 2θ = 20.3°, 21.5°, 22.9°, 26.4°, and 27.9°. These peaks correspond to the (020), (100), (021), (101), (111), (121), and (040) planes, respectively, indicating an orthorhombic crystal structure [18,33,43].

XRD analysis over time aims to track the evolution of the material’s degree of crystallinity during aging. It is well-known that high material crystallinity results in a high elastic modulus and low elongation at rupture [19,20]. Additionally, the brittleness of P3HB is also caused by an aging process at room temperature, which is induced by crystalline evolution over time, thereby restricting the mobility of the amorphous fraction of the material [32]. 

**Figure 4:**
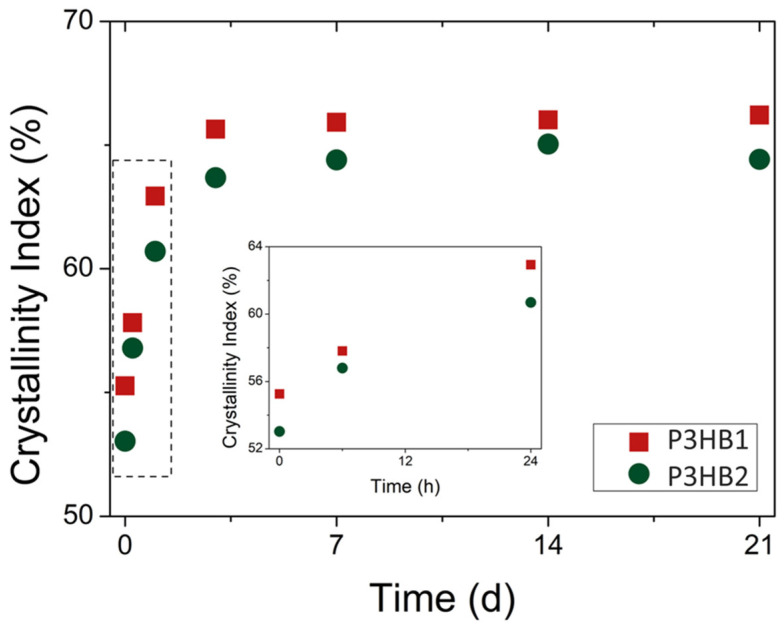
*X_XRD_* as a function of aging time at room temperature.

*X_XRD_* values as a function of aging time are shown in Figure 4. To mitigate the effects of crystallinity variation with sample depth relative to its surface [44], XRD measurements were conducted at approximately the same central position within the specimen. The crystallinity of the samples obtained at time 0 h (immediately after the molding process, with a logistic time between obtaining and performing the analysis of up to 20 min) was 55.3% for P3HB1 and 53% for P3HB2. An increase in the crystallinity of the samples was notable during the aging time after the injection process, with a more significant increase occurring within the first 24 h, as shown in detail in Figure 4, where there is an almost linear relationship of crystallinity increase. This behavior is similar to what has been reported in the literature for P3HB [45]. At the 24 h time point, *X_XRD_* values of 62.9% and 60.7% were observed for P3HB1 and P3HB2, respectively. The crystallization values tend to stabilize after 72 h in both materials. Beyond this time, a plateau of crystallinity is established over the aging time, with average *X_XRD_* values of 66% and 64.6% for P3HB1 and P3HB2, respectively. These values are consistent with those observed by *X_DSC_* for the unprocessed samples. De Koning and Lemstra [46] observed a similar pattern during the aging of P3HB at 25 °C, using dilatometry data that were subsequently converted into crystallinity. They reported a progressive increase in P3HB crystallinity, from 56% (as molded) to 63% after 200 h, reaching a crystallinity of 65% after one month of storage.

Although there is a significant increase in crystallinity over the aging time, it is interesting to note, based on the diffraction patterns (Figure 3), that there was no change in the crystalline structure of the two studied P3HBs. In a similar context, the rise in crystallinity was attributed to secondary crystallization rather than a crystal rearrangement [46]. Kurusu et al. [45] evaluated the aging of P3HB over a period of 21 days at room temperature. Based on the SAXS diffraction patterns, they did not observe any alteration in the position of the maximum scattering peak, which is reflected in the unchanged long period of the crystal. Thus, as an effect of the aging time, they noted an increase in the lamellar thickness of the crystal along with a reduction in the thickness of the interlamellar amorphous phase, while maintaining a constant long period of the crystal.

### 3.4. Mechanical Properties

There is a linear relationship between the crystallinity and the elastic modulus of P3HB. As the aging time increases, the material tends to become more brittle due to increased crystallinity [32,45,47]. De Koning and Lemstra [46] attributed P3HB fragility to a process of secondary crystallization, i.e., the progressive increase in crystallinity that effectively confines amorphous chains between the crystals. As a consequence, the material becomes more fragile. Subsequently, Cretóis et al. [32] demonstrated that, in addition to the secondary crystallization process, there is also the contribution of physical aging at room temperature, further contributing to P3HB embrittlement.

**Figure 5:**
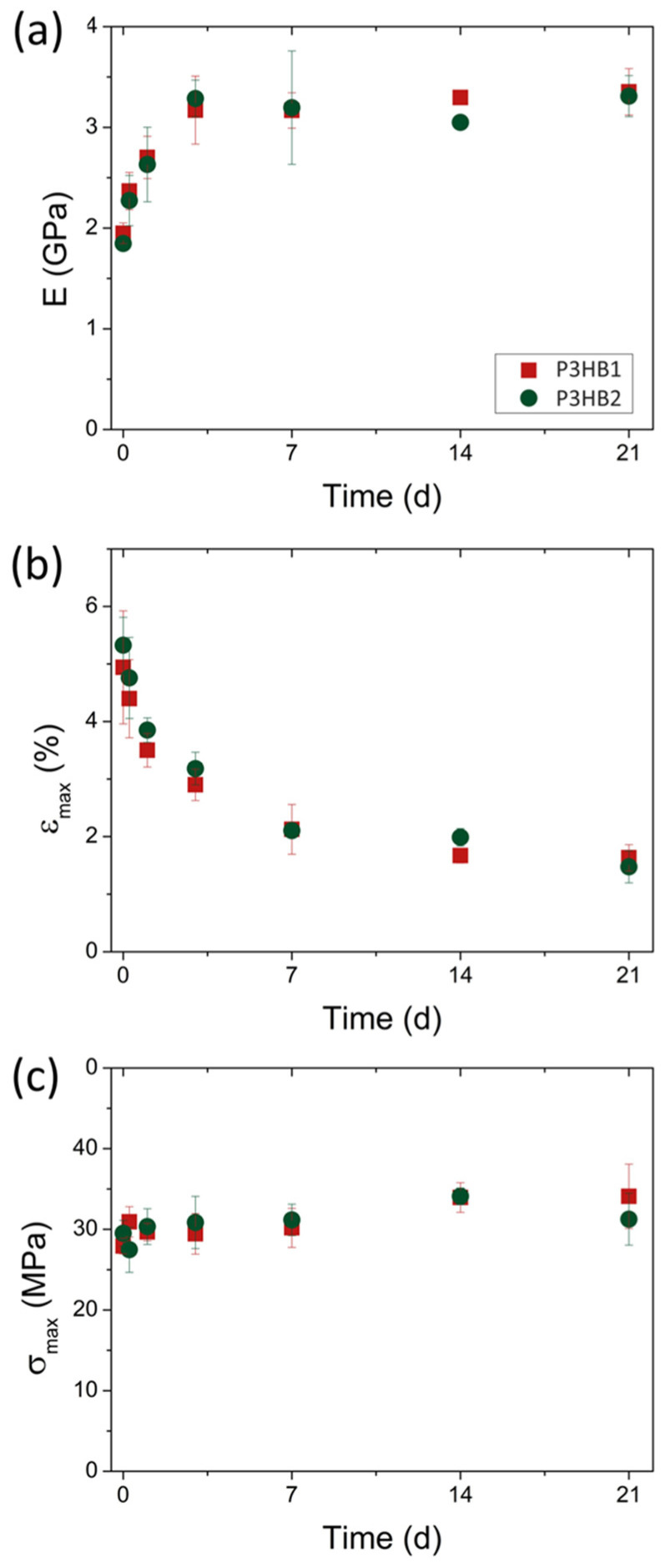
Mechanical properties as a function of aging time at room temperature. (**a**) E, (**b**) ε_max_, and (**c**) σ_max_.

Therefore, tensile tests were conducted at the same analysis times as XRD to evaluate the impact of aging on the mechanical properties (Figure 5). Mechanical results showed trends in behavior that corroborate the aging effect on the samples. As expected, E (Figure 5a) and ε_max_ (Figure 5b) exhibit inversely proportional trends, while σ_max_ (Figure 5c) remained unaffected by the aging process. 

E increases with aging time due to the increased crystallinity of the P3HBs, demonstrating behavior similar to that observed in *X_XRD_* with stabilization after 72 h, establishing a plateau in the elastic modulus thereafter. On the other hand, the increase in crystallinity caused a decrease in ε_max_. However, the plateau in ε_max_ occurs at a longer time, starting from 7 days of aging. One possible cause is the greater sensitivity of deformation caused by the increased mobility restriction of the amorphous phase of P3HB during the aging time.

It is worth mentioning that, despite the different routes of obtaining P3HBs, both exhibit similar physical properties. This behavior can be attributed to their high and similar molecular weight (M_w_) values, which prove to be a determining factor in P3HB properties [31]. Furthermore, the obtained P3HBs exhibit a high elastic modulus and low elongation at break, directly correlated with the material’s crystallinity. These characteristics of a stiffer material suggest the utilization of P3HBs in bone-related applications rather than in soft tissue engineering applications [22].

## 4. Conclusions

Based on the results obtained in this study, it can be concluded that the investigated P3HBs demonstrate remarkable similarity and comparability in their thermal and mechanical properties. A consistent embrittlement behavior was observed over the aging period, associated with an increase in crystallinity. These biopolymers, with their physical properties intrinsically related to the molecular weight, manifest features that could be fundamental for their practical suitability.

It is important to emphasize that the obtained results align with the physical properties of standard P3HBs reported in the literature. This consistency reinforces the viability and robustness of the P3HBs synthesized in this study, highlighting the potential of these biopolymers to expand the scope of biomaterials used in bone tissue engineering and related fields.

Furthermore, when comparing CO_2_ and CH_4_ as raw materials, both prove highly attractive due to their scalability, widespread availability, and favorable methanotroph growth rates. Both processes require specialized fermenters, with cyanobacteria necessitating a photobioreactor and methanotrophs requiring a continuous-flow fermenter. Subsequent optimization is imperative to minimize losses and reduce production costs. Considerations such as the recycling of the fermentation medium and the valorization of residual biomass for protein extraction or biogas production are crucial for energy efficiency and sustainability.

As PHA materials become more widely available in the market, there is an expected increase in interest in this class of polymers, driving the development of successful benchmark products and advancing the replacement of fossil polymers.

## Figures and Tables

**Table 1:** Main characteristics of P3HBs.

	P3HB1	P3HB2
Host	Cyanobacteria (pure culture)	Aerobic methanotrophs (pure culture)
Strain	*Synechocystis* sp. PCC 6714	*Methylocystis* sp. GB 25
Carbon source	CO_2_	CH_4_
Appearance	White powder	White powder

**Table 2:** P3HBs characteristics molecular weights.

	P3HB1	P3HB2
M_n_ (Da)	696,394	207,475
M_w_ (Da)	1,073,000	1,084,000
M_w_/M_n_	1.54	5.23

**Table 3:** Thermal properties obtained via DSC. T_g_, T_c_, T_m_, and *X_DSC_*.

Thermal Properties	P3HB1	P3HB2
T_g_ (°C)	4.02	4.43
T_c_ (°C)	79.56	70.29
T_m_ (°C)	174.32	175.10
*X_DSC_* (%)	64.49	63.19

## Data Availability

The data presented in this study are available in this article.
